# Cellular sentinels: empowering survival and immune defense in hematopoietic stem cell transplantation through mesenchymal stem cells and T lymphocytes

**DOI:** 10.1186/s12916-025-03987-2

**Published:** 2025-03-18

**Authors:** Tzong-Shyuan Tai, Yun-Hsiang Chen, Chao-Ling Yao, Jiun-Han Lin, Yu-Shao Yang, Jai-Wen Shi, Li-Wen Fang, Duen-Wei Hsu, Shu-Chen Kuo, Shu-Ching Hsu

**Affiliations:** 1 Department of Medical Research and Development, Chang Gung Memorial Hospital, Taoyuan, 33305 Taiwan; 2 Department of Life Science, Fu-Jen Catholic University, New Taipei City, 242062 Taiwan; 3 Center for Neuropsychiatric Research, National Health Research Institutes, Miaoli, 35053 Taiwan; 4 Department of Chemical Engineering, National Cheng Kung University, Tainan City, 70101 Taiwan; 5 Department of Industrial Technology, Ministry of Economic Affairs, Taipei, 100210 Taiwan; 6 Food Industry Research and Development Institute, Hsinchu, 30062 Taiwan; 7 National Institute of Infectious Diseases and Vaccinology, National Health Research Institutes, 35 Keyan Road, Zhunan, Miaoli 35053 Taiwan; 8 Institute of Bioinformatics and Structural Biology, National Tsing Hua University, Hsinchu, 30013 Taiwan; 9 Department of Biotechnology, National Kaohsiung Normal University, Kaohsiung City, 82444 Taiwan; 10 Department of Nutrition, I-Shou University, Kaohsiung City, 82445 Taiwan; 11 Immunology Research and Development Center, China Medical University, Taichung City, 404328 Taiwan; 12 Department of Biomedical Sciences and Engineering, Tzu Chi University, Hualien, 97004 Taiwan; 13 Graduate Institute of Medicine, College of Medicine, Kaohsiung Medical University, Kaohsiung City, 80761 Taiwan; 14 Doctoral Program in Tissue Engineering and Regenerative Medicine, National Chung Hsing University, Taichung City, 40227 Taiwan

**Keywords:** Hematopoietic stem cell transplantation (HSCT), Engraftment enhancement, Mesenchymal stem cells (MSCs), Activated T cells, Neutrophil regeneration

## Abstract

**Background:**

Hematopoietic stem cell transplantation (HSCT) is a critical treatment for hematologic disorders such as leukemia, lymphoma, and specific immune deficiencies. Despite its efficacy, challenges such as engraftment failure and delayed neutrophil regeneration remain significant barriers. These complications lead to prolonged cytopenia, increased risks of infections and other complications, and elevated morbidity and mortality rates. While mesenchymal stem cells (MSCs) are known to play essential roles in supporting hematopoiesis, the precise mechanisms and interactions between MSCs and other cellular components in HSCT require further investigation.

**Methods:**

To address these challenges, we explored the combined infusion of allotype-cord blood hematopoietic stem cells (HSCs) and activated T cells from the same donor along with third-party MSCs. The study assessed the effects of this triple-cell therapy on neutrophil differentiation and function ex vivo and in vivo. Using a respiratory infection model, we evaluated the accumulation of human neutrophils, cytokine secretion (IL-6 and IL-8), bacterial clearance, and overall survival compared to control groups.

**Results:**

The triple-cell therapy demonstrated a significant improvement in the differentiation of human HSCs into neutrophils both in ex vivo and in vivo. In the respiratory infection model, this approach resulted in enhanced accumulation of human neutrophils, increased secretion of IL-6 and IL-8, superior bacterial clearance, and reduced mortality rates compared to the control group. These findings highlight the synergistic interplay between allo-HSCs, MSCs, and activated T cells in promoting neutrophil production and function.

**Conclusions:**

Our study presents a novel therapeutic strategy combining allo-HSCs, activated T cells, and third-party MSCs to enhance neutrophil production and functionality post-transplantation. This approach not only accelerates neutrophil regeneration but also improves resistance to infections, offering a promising avenue to overcome engraftment challenges in HSCT.

## Background

Hematopoietic stem cells (HSCs) are rare, multipotent cells residing in the bone marrow, responsible for generating all blood and immune cells [1]. Over the years, significant insights into HSC biology have been gained through the study of these rare populations, genetic manipulation, and advanced animal model imaging. HSC function is regulated by both intrinsic and extrinsic signals, which control critical processes such as self-renewal, quiescence, proliferation, and multilineage differentiation, ensuring their lifelong contribution to an individual’s hematopoietic system [2]. Since their discovery in 1961, HSCs have played a pivotal role in clinical bone marrow transplantation, providing potentially curative treatments for a range of hematological disorders and cancers. These transplants restore hematopoiesis in patients affected by myeloablative radio/chemotherapy or those with defective blood cell production or function [3]. This revolutionary therapy has transformed the management of once-fatal malignant and non-malignant blood disorders, offering lifesaving treatments to patients. However, despite substantial progress, the intricate mechanisms governing HSC behavior remain incompletely understood, making this area a vital focus of ongoing biomedical research.

The bone marrow microenvironment is a highly sophisticated and finely regulated system that plays a critical role in maintaining HSC self-renewal and multilineage differentiation. Mesenchymal stem/stromal cells (MSCs) are a key component of the HSC niche within the bone marrow [4–6]. These cells are heterogeneous and possess a remarkable ability to differentiate both in vitro and in vivo, contributing to the development and maintenance of mesenchymal tissues, while also exhibiting potent immunomodulatory properties [7, 8]. MSCs have garnered significant attention as a therapeutic modality due to their inherent immunosuppressive and anti-inflammatory characteristics. Ongoing preclinical and clinical investigations suggest that MSCs could revolutionize the management of graft-versus-host disease (GvHD) and autoimmune disorders. These pioneering studies indicate that MSCs have the potential to improve outcomes in cell or organ transplantation by reducing rejection and potentially eliminating the need for extended immunosuppressive therapy [9, 10].

Neutropenia, defined as an abnormally low number of neutrophils, is a major concern for cancer patients undergoing chemotherapy, recipients of hematopoietic stem cell transplants (HSCT), and individuals receiving chimeric antigen receptor (CAR) T-cell therapy [11, 12]. The intensive preparative regimens used prior to transplantation can temporarily impair bone marrow function, leaving patients immunocompromised until the transplanted stem cells engraft. The severity and duration of neutropenia are influenced by the intensity of the preparative regimen and the graft source, with cord blood transplants often associated with prolonged neutropenia [13, 14]. Similarly, both chemotherapy and CAR-T therapy induce neutropenia and lymphopenia, which increases the risk of life-threatening infections in cancer patients, potentially leading to treatment interruptions and reduced therapeutic efficacy [15]. Neutropenic fever and respiratory infections significantly contribute to morbidity and mortality [16], underscoring the urgent need for novel strategies to mitigate these risks. While antimicrobial prophylaxis and growth factor support offer partial protection, achieving the optimal balance between immunosuppression for tumor cytotoxicity or engraftment and the accompanying infection risk remains a considerable challenge [17]. Managing opportunistic infections arising during treatment is crucial to optimizing therapeutic outcomes.

In our previous study, we discovered that MSCs specifically stimulate interleukin-17 (IL-17) production in activated CD4^+^CD45RO^+^ memory T cells through direct cell–cell interaction, without affecting other T cell subsets. The IL-17 produced subsequently enhances neutrophil phagocytic activity, highlighting how MSCs can coordinate innate immunity by reactivating adaptive CD4^+^ memory T cells [18]. Building on this finding, our current research reveals the synergistic effect of T cells and MSCs in promoting the engraftment and reconstitution of umbilical cord blood-derived HSCs in immunodeficient mice. This synergy leads to increased neutrophil levels and significantly improved survival rates, further emphasizing the therapeutic potential of this novel approach.

## Methods

### Mice

NOD.CB17-Prkdc^scid^/Tcu (NOD/SCID) and NOD/Shi-scid IL2Rgamma(null) (NOG) mice were bred and maintained in the Laboratory Animal Center of the National Health Research Institutes, Miaoli, Taiwan. Four-week-old mice served as recipients for this study. Mice were housed under controlled conditions at a constant temperature of 25 °C, with ad libitum access to food and water. The light cycle was set to 12 h of light and 12 h of darkness. All animal experiments were performed in compliance with protocols approved by the Institutional Animal Care and Use Committee of the National Health Research Institutes (NHRI-IACUC-100034-A, NHRI-IACUC-100126-A, NHRI-IACUC-102011-A, NHRI-IACUC-108110A, NHRI-IACUC-108039-AE, NHRI-IACUC-108110M2-AE-S01).

### Culture of human MSCs and human HSCs

Human bone marrow-derived MSCs (#PT-2501) were purchased from Lonza Walkersville Inc., all of which met Lonza’s quality standards. Human CD34^+^ umbilical cord blood HSCs were either purchased from Lonza Walkersville Inc. (#2C-101H) or isolated from human umbilical cord blood donated by volunteers using the CD34^+^ Progenitor Cell Isolation Kit (Miltenyi Biotec, Inc., Auburn, CA, USA) according to the manufacturer’s instructions. Human MSCs were cultured in an expansion medium consisting of Iscove’s modified Dulbecco’s medium (IMDM, Sigma-Aldrich, St. Louis, MO, USA) and 10% fetal bovine serum (FBS, Hyclone, Logan, UT, USA), supplemented with 10 ng/mL bFGF (R&D Systems, Minneapolis, MN, USA), 100 U/mL penicillin, 100 μg/mL streptomycin [19], and 2 mM L-glutamine (Sigma-Aldrich). Human CD34^+^ HSCs were cultured in Dulbecco’s modified Eagle’s medium (DMEM, Invitrogen, Grand Island, NY, USA) supplemented with 10% FBS and antibiotics, as described above for BM-MSCs. Approval from the institutional review board of Taoyuan General Hospital, Ministry of Health and Welfare, Taiwan (TYGH100040, TYGH109026) and the Research Ethics Committee of National Health Research Institutes (EC1000802, EC1001101-R1, EC1030604-E, EC1111003-W) were obtained prior to the commencement of this study.

### Preparation of human anti-CD3/CD28 activated T lymphocytes

Human T lymphocytes were isolated from umbilical cord blood autologous samples, using Ficoll-Paque density gradient centrifugation. Subsequently, CD3^+^ T cells were purified using a human Pan T cell isolation kit (Miltenyi Biotec), achieving a purity exceeding 95%. The purified CD3^+^ T cells were then activated with human T-activator CD3/CD28 Dynabeads (Invitrogen) for 24 h before being collected for further use. All procedures involving human immune cells were conducted in compliance with the approved guidelines of the Research Ethics Committee at the National Health Research Institutes (Approval numbers: EC1000802, EC1030604-E, EC1111215-W).

### Generation of humanized mice

CD34^+^ HSCs were isolated through positive immunomagnetic selection following the manufacturer’s instructions (Miltenyi Biotec, Inc.). Various cell combinations, including CD34^+^ HSCs alone (5 × 10^4^), HSCs/T cells (5 × 10^4^: 5 × 10^4^), HSCs/MSCs (5 × 10^4^: 2 × 10^4^), and HSCs/MSCs/T (5 × 10^4^: 2 × 10^4^: 5 × 10^4^), were intravenously infused into 4-week-old NOD/SCID mice after sub-lethally pre-irradiation (2000 mGy) using a RS2000 X-ray biological irradiator (Rad Source Technologies, Inc., Alpharetta, GA). Four weeks post-transplantation, blood samples were collected from each human HSC-infused mouse via venous puncture and analyzed either by staining for human-specific surface markers (hCD45) or screening for human Alu signals by real-time PCR.

### HSC ex vivo differentiation and in vivo reconstitution

CD34^+^ HSCs (1 × 10^5^) were cultured either alone or in combination with 2 × 10^4^ BM-MSCs and 1 × 10^5^ autologous activated T cells for 14 days. The cells were collected and surface stained with anti-human CD11b-APC, anti-human CD3-PerCP, and anti-CD105-PE-Cyanine7 antibodies (BioLegend), followed by intracellular labeling with an anti-myeloperoxidase (MPO)-FITC antibody following the manufacturer’s protocols (BioLegend). Data analysis involved gating out CD3- and CD105-double negative cells as HSC differentiated cells, followed by FACS analysis to determine CD11b^+^MPO^+^ expression levels. For in vivo HSC reconstitution analysis, bone marrow cells from the various experimental groups of humanized mice (12 to 16 weeks post cells transplantation) were collected and stained with anti-human CD45-PE (eBioscience), anti-mouse CD45-APC (BioLegend, San Diego, CA, USA), and anti-human MPO-FITC antibodies. FACS analysis was conducted to evaluate the frequency of human CD45^+^ and human MPO-expressing cells.

### Phagocytosis assay

Human peripheral blood mononuclear cells (PBMCs), neutrophils, and 14-day co-cultures of human CD34^+^ HSCs (1 × 10^5^ cells, derived from three different donors), BM-MSCs (2 × 10^4^ cells), and activated Pan T cells (1 × 10^5^ cells) were harvested. There cells were treated with 100 µg/mL pHrodo™ Red Zymosan A BioParticles™ Conjugate (Invitrogen) in IMDM supplemented with 10% FBS for 2 h. The frequency of the phagocytotic population (pHrodo-Red^+^) was analyzed using flow cytometry. The CD11b^+^MPO^+^ population was identified as neutrophils derived from HSC-MSC-T cell (HMT) co-cultures.

### RNA isolation and real-time PCR analysis

After 14-day co-culture, suspended cells were harvested and washed three times with 1 × DPBS (Gibco). Total RNA was extracted using the Trizol reagent (Invitrogen) and converted to cDNA using a ReverTra Ace set (Toyobo Life Science) according to the manufacturer’s instructions. Real-time PCR was performed using an ABI Prism 7900 system (Applied Biosystems). For each sample, the cycle threshold (Ct) value was determined. The results were normalized to the levels of the GAPDH (glyceraldehyde-3-phosphate dehydrogenase) gene on the same plate. The level of mRNA expression for different cell groups was calculated using the 2ΔCt method. Primers specific for each gene were designed as follows:

Human GAPDH, GAGTCAACGGATTTGGTCGT (forward primer, F), TTGATTTTGGAGGGATCTCG (reverse primer, R); IL-17 A, CCGCCACTTGGGCTGCATCA (F), GGGCAGTGTGGAGGCTCCCT (R); S100 calcium binding protein A8 (S100A8), GGCAAGTCCGTGGGCATCATGT (F), CAGGTCATCCCTGTAGACGGCA (R); MPO, GGACCACGGCCTCCCAGGAT (F), GGTTCCTCAGCACCGTGCCC (R); GATA1: AACCGGCCACTGACCATGCG (F), CCTTCGGCTGCTCCTGTGCC (R); TMF1: AGGGGAGCGAAGCCGTTCCT (F), TCATCGCCCCTCCTCAGCCG(R); IL7R: GCACGCTGCCCCCTCCATTT (F), GGCTGACCCTGAGCAACTGGG (R); NOX2: CTCTGAACTTGGAGACAGGCAAA (F), CACAGCGTGATGACAACTCCAG (R); LCN2: ACGGGAGAACCAAGGAGCTGAC (F), TGGGACAGGGAAGACGATGTGG (R).

### Enzyme-linked immunosorbent assay (ELISA)

Mouse serum was collected 1 day after *Pseudomonas aeruginosa* infection. Human IL-6 and IL-8 levels were then measured using commercial ELISA kits, following the manufacturer’s protocols (eBioscience).

### Pulmonary infection model and bacterial CFU assessment

The pulmonary infection model was adapted from a previous study with modifications [20]. A bacterial suspension was prepared by mixing with mucin derived from porcine stomach (type 3; Sigma-Aldrich, Saint Louis, USA) to achieve a final concentration of 5% mucin. Mice were anesthetized using isoflurane to minimize discomfort and distress. To induce pneumonia, a sublethal dose of *Pseudomonas aeruginosa* (LD50 to be approximately 3 × 10⁶ colony-forming units (CFUs)) was administered via intra-tracheal (IT) insertion using a 24-gauge feeding needle. At 24 h post-infection, half of the lung tissues were harvested, rinsed with cold HBSS, and homogenized using a tissue chopper. The lung homogenate was subjected to serial log₁₀ dilutions, and aliquots of each dilution were plated onto Luria-Bertani (LB) agar plates. Plates were incubated overnight at 37 °C, and bacterial CFUs were quantified.

### Isolation of lung interstitial cells

Half of the lung tissues were rinsed with cold HBSS and sectioned into 500-µm slices. The samples were incubated in HBSS supplemented with 10% FBS, DNase (75 µg/mL; Sigma-Aldrich), and Collagenase D (1 mg/mL; Roche) for 30–60 min at 37 °C. Following digestion, the resulting cell suspension was filtered through a sterile filter, washed with PBS, and stained with anti-human CD45-PE (eBioscience), anti-mouse CD45-APC (BioLegend, San Diego, CA, USA), and anti-human MPO-FITC antibodies. Flow cytometry analysis was performed to determine the frequency of human CD45^+^ and human MPO-expressing cells.

### Fluorescence-activated cell sorting (FACS) analysis

Cells were harvested and washed once with FACS buffer (PBS containing 1% FBS) to prevent non-specific binding, then stained with fluorochrome-conjugated antibodies specific to the indicated surface markers for 30 min on ice. After incubation, cells were washed three times with FACS buffer to remove unbound antibodies, resuspended, and analyzed using a Cytek Aurora flow cytometer (Cytek). Appropriate compensation was included to ensure accurate gating and marker detection. Data were analyzed using FlowJo software (version 10.8.0) following standard gating strategies to exclude debris and dead cells, with fluorescence signals adjusted based on internal controls. The antibody clones and their corresponding fluorochromes are listed below: anti-human CD45-BV421 (Clone: HI30, BioLegend, Cat#304,032); anti-human CD11b-APC (Clone: M1/70, BioLegend, Cat#101,212); anti-human CD19-BV510 (Clone: SJ25C1, BD Biosciences, Cat#562,947); anti-human CD3-PerCP (Clone: OKT3, BioLegend, Cat#317,338); anti-human MPO-FITC (Clone: MPO421-8B2, BioLegend, Cat#347,201).

### Statistical analyses

All graphs were generated, and statistical analyses were performed using GraphPad Prism (versions 5.02 and 8.0.1). Survival rate data were analyzed using the Log-rank test, while mRNA expression data were evaluated using ANOVA followed by the Bonferroni post-test. Differences with a *p*-value of less than 0.05 were considered statistically significant.

## Results

### Synergistic role of MSCs and T cells in directing HSC differentiation toward specialized immune lineages

**Fig. 1:**
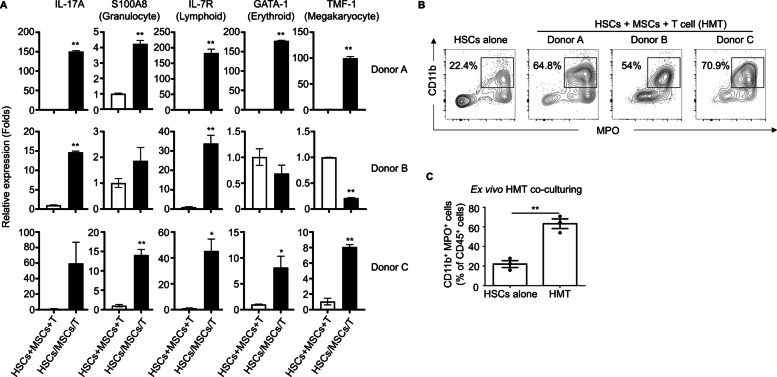
Illustrates the guidance of human hematopoietic stem cell (HSC) differentiation toward specialized immune lineages with human bone marrow-derived mesenchymal stem cells (BM-MSCs) and CD3^+^ T cells. **A** Human CD34^+^HSCs (1 × 10^5^) were isolated from three donors and cultured alone or co-cultured with allogenic MSCs (2 × 10^4^) and activated stem cell donor-matched T cells (1 × 10.^5^) (HSCs/MSCs/T) for 14 days ex vivo. After that, cells were harvested, and their total RNA was extracted to examine the expression of specific genes using quantitative real-time PCR. RNA from HSCs alone, MSCs alone, and T cell alone was evenly mixed in equal amounts as the control group sample, HSCs + MSCs + T. The expression levels of representative genes, including S100A8 (Granulocyte lineage), IL-7R (Lymphoid lineage), GATA-1 (Erythroid lineage), and TMF-1 (Megakaryocyte lineage), are depicted as folds of the control level. Suspensive HSC cells alone and the combination of (HSCs + MSCs + T) from three various HSC donors were harvested at 14 days, and the CD11b⁺ MPO⁺ population was identified using flow cytometry (**B**). The statistical analysis of ex vivo differentiation from three different donors is summarized in **C**. Data were expressed as mean ± S.E.M., and significant differences between groups were indicated (*p < 0.05, **p < 0.01; two-sided unpaired *t* test, ns, not significant)

Our previous study demonstrated the significant impact of bone marrow-derived MSCs on modulating neutrophil activation through the regulation of interleukin-17 (IL-17) secretion by memory CD4^+^ T cells [18]. Given the established role of IL-17 in granulopoiesis [21, 22], we further investigated the potential of MSCs, both alone and in combination with activated T cells, to enhance HSC differentiation—a critical process for immune system functionality. To address potential variations in baseline gene expression due to different cellular backgrounds, RNA was separately extracted from HSCs, MSCs, and T cells. These RNA samples were then combined in equal proportions to serve as control groups (HSCs + MSCs + T) for comparison with the co-cultured samples (HSCs/MSCs/T). A significant increase in IL-17A expression was observed in the co-cultured groups (Fig. 1A), consistent with our previous findings [18]. Additionally, co-culturing enhanced the expression of key lineage markers, including S100 calcium-binding protein A8 (S100A8, granulocyte lineage), IL-7R (lymphoid lineage), and the transcription factors GATA-1 (erythroid lineage) and TMF-1 (megakaryocyte lineage) within the HSC population (Fig. 1A). These findings highlight the potential of co-culturing HSCs with MSCs and T cells to promote HSC differentiation into various immune cell lineages. To further delineate the neutrophil phenotype, we established an ex vivo co-culture system comprising HSCs, MSCs, and T cells. HSCs cultured alone exhibited approximately 22.4% neutrophil differentiation, identified as CD11b^+^MPO^+^ cells. In contrast, co-culture with MSCs and T cells enhanced neutrophil differentiation to approximately 63.2% (Fig. 1B and C). Statistical analysis using a two-tailed unpaired *t*-test showed a highly significant difference between the HSC-alone and HMT groups (*p* = 0.0024). This finding highlights the united effect of MSCs and T cells in promoting the differentiation of HSCs into neutrophils.

**Fig. 2:**
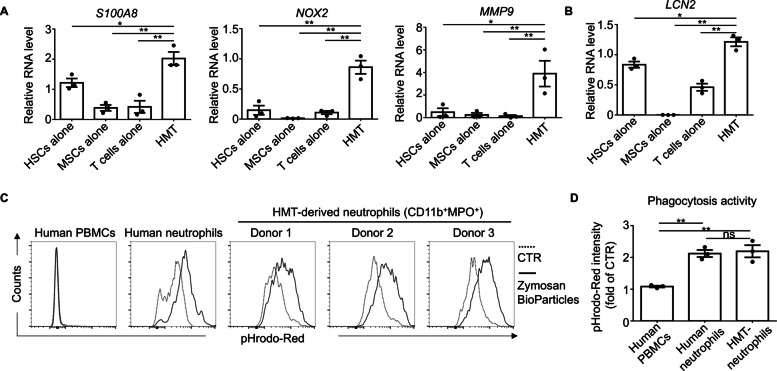
Co-culture of human CD34^+^ HSCs with human MSCs and autologous T cells enhances granulocytic lineage development and phagocytic function. Human CD34^+^ HSCs were cultured alone or co-cultured with BM-MSCs and autologous CD3^+^ T cells at defined ratios (HSCs: 1 × 10⁵ cells, T cells: 1 × 10⁵ cells, MSCs: 2 × 10⁴ cells) with 4 × 10³ CD3/CD28 T-cell activator beads for 14 daysin vitro. Total RNA was extracted from cultured cells and analyzed by real-time PCR to quantify mRNA expression of neutrophil-associated markers, including *S100A8*,* NOX2*,* MMP9*, and* LCN2*(panels **A**, **B**). Phagocytic function was assessed by co-culturing suspension cells with 100 µg/ml pHrodo-red bioparticles for 2 h, with subsequent analysis of the CD11b⁺ MPO⁺ cell population (panel **C**). Phagocytic activity was determined across human peripheral blood mononuclear cells (PBMCs), freshly isolated neutrophils, and hematopoietic microenvironment (HMT)-derived CD11b⁺ MPO⁺ cells. Data are presented as means ± standard error of the mean (S.E.M.) from three independent experiments. Statistical significance was assessed using a two-sided unpaired *t* test (*p < 0.05, **p < 0.01, ns = not significant)

Further analysis revealed that while S100A8 expression was observed in HSCs alone, the combination of HSCs, MSCs, and T cells (HMT) exhibited increased expression of key markers associated with neutrophil function, including S100A8, NADPH oxidase 2 (NOX2), and Matrix metalloproteinase-9 (MMP-9), within the differentiated cell population (Fig. 2A). Lipocalin-2 (LCN2), also known as neutrophil gelatinase-associated lipocalin (NGAL), is a protein encoded by the *LCN2* gene in humans. It plays a pivotal role in innate immunity by sequestering iron, thereby limiting bacteria growth. As shown in Fig. 2B, LCN2 expression was detected in both the HSC-alone and T cell-alone groups, consistent with previous studies [22, 23]. Notably, LCN2 expression was significantly elevated in the HMT group compared to HSCs alone (*p* = 0.0135), MSCs alone (*p* < 0.0001), and T cells alone (*p* = 0.0014, unpaired two-tailed *t*-test) (Fig. 2B). These findings suggest that co-culturing HSCs with MSCs and activated T cells may enhance anti-bacterial activity.

Furthermore, the phagocytic activity of HMT-derived neutrophils was comparable to that of freshly isolated human neutrophils, indicating that these cells possess normal functional capabilities (Fig. 2C and D). In contrast, human peripheral blood mononuclear cells (PBMCs) showed no phagocytic activity and served as a negative control group. In summary, co-culturing HSCs with MSCs and activated T cells markedly enhances neutrophil differentiation and supports the generation of functionally competent neutrophils.

### Enhanced survival rates in mice receiving human HSCs with the addition of MSCs and activated T cells

**Fig. 3:**
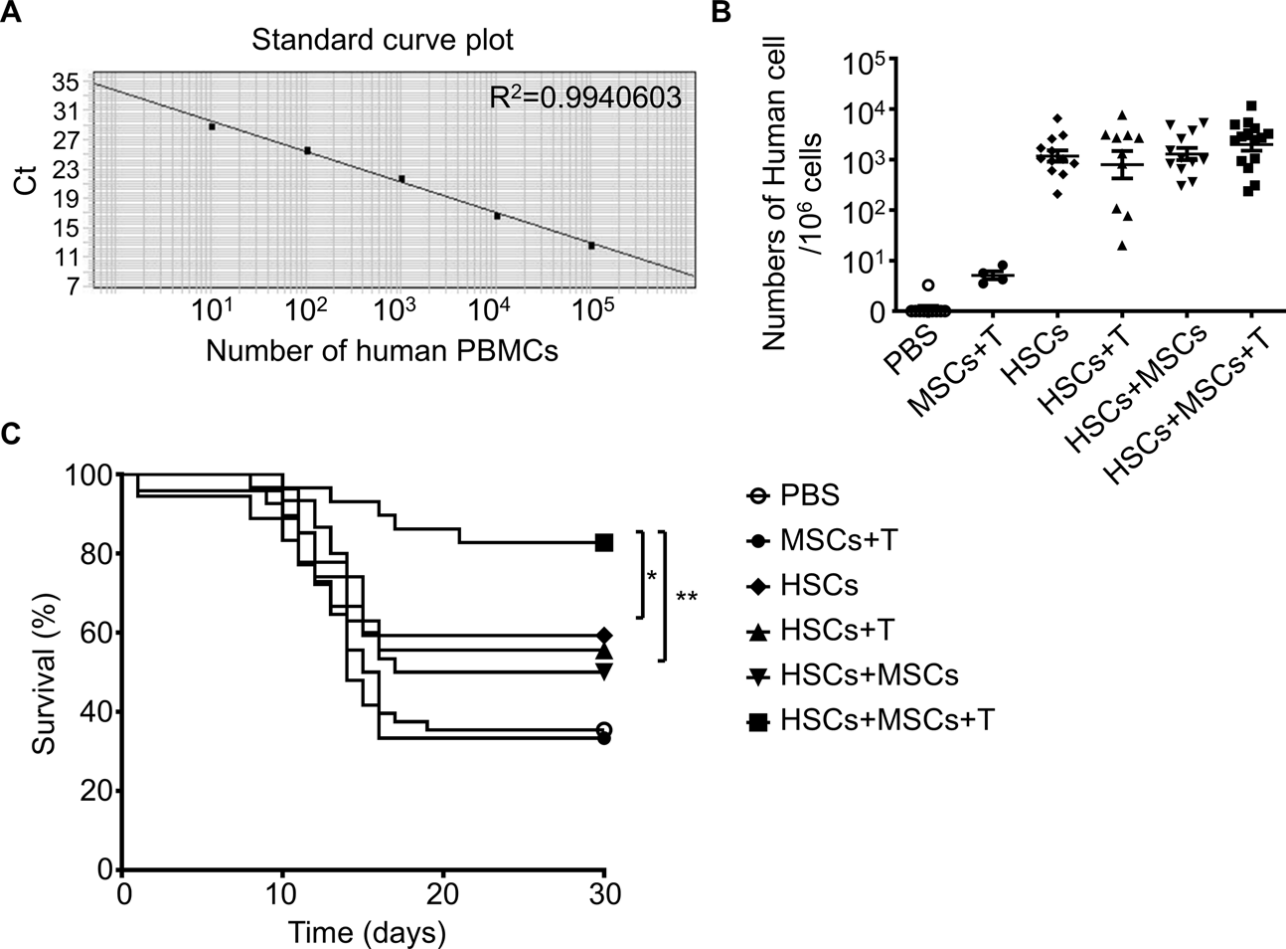
Co-transplantation of human HSCs with human MSCs and T cells enhanced survival of irradiated NOD/SCID mice. **A** A standard curve for the calculation of human cell numbers. Human peripheral blood mononuclear cells (PBMCs) were tenfold serially diluted (10^6^, 10^5^, 10^4^, 10^3^, 10^2^, 10^1^, 10^0^ cells) and mixed with mouse splenocytes to a total cell number of 1 × 10^6^. Each mixture was subjected to genomic DNA extraction for the real-time PCR analysis to quantify human Alu repeat sequence and mouse albumin. A standard curve is presented as a semi-log regression line plot of mean Ct values versus the logarithm of input human PBMC numbers. **B** Measurement of human-derived cells in irradiated NOD/SCID mice after transplantation. NOD/SCID mice (4-week-old) were given 2000 mGy irradiation and then received an intravenous infusion of PBS or various combinations of human HSCs (1 × 10^5^), MSCs (5 × 10^5^), and autologous T cells (1 × 10.^5^) as indicated (PBS: ○, *n* = 10; MSCs + T: ●, *n* = 4; HSCs: ◆, *n* = 15; HSCs + T: ▲, *n* = 14; HSCs + MSCs: ▼, *n* = 14; HSCs + MSCs + T: ■, *n* = 23). After 4 weeks of transplantation, the peripheral blood of recipient mice was analyzed for the content of human Alu repeat sequence by real-time PCR, and the number of human-derived cells was calculated from the Ct value using the standard curve mentioned above. Data were expressed as mean ± S.E.M., and significant differences between groups were indicated (*p < 0.05, **p < 0.01; one-way ANOVA). **C** Survival curves of irradiated NOD/SCID mice after transplantation. After cell transplantation, the survival of recipient mice was followed up for 30 days. Survival curves were analyzed for the differences between groups using the Log-rank test, and significant differences were indicated (*p < 0.05, **p < 0.01)

To validate our hypothesis in a living system, we conducted an in vivo study by transplanting human HSCs alone or in combination with human BM-MSCs and/or activated human T cells into irradiated 4-week-old NOD-SCID mice via tail vein injection. Control groups received either phosphate-buffered saline (PBS) or MSCs combined with activated T cells. Across different batches of animal preparations, significant variability in the reconstitution efficiency of HSCs was observed, even within the same cell treatment group. To minimize variability between batches and individuals, we employed qPCR to establish an absolute quantification method for the relative signal of human PBMCs in each mouse. To accurately assess human cell reconstitution within murine hosts, a standard curve was established using quantitative PCR to correlate the human Alu signal with the number of human immune cells (Fig. 3A). This method enhances the sensitivity of detecting human blood cell reconstitution in mice compared to flow cytometry staining, allowing us to detect as few as 10 human cells in the recipient mice at 4 weeks post cell infusion (Fig. 3B). As expected, minimal human cells were detected in the PBS control group, whereas mice receiving MSCs and activated T cells showed a modest reconstitution of approximately 10 human cells per one million cells examined. Transplantation of HSCs alone resulted in a significant increase in human cell reconstitution, with approximately 1000 human cells per one million cells examined. Furthermore, mice receiving HSCs with activated T cells, HSCs with MSCs, or HSCs with both MSCs and activated T cells displayed comparable levels of human cell reconstitution (Fig. 3B).

The critical measure of success was post-transplant survival rates. In the PBS group, the survival rate was low at 35.4% (17 out of 48 mice), and the addition of MSCs and activated T cells did not improve survival, with a rate of 29.4% (5 out of 17 mice). Notably, transplantation of HSCs alone significantly improved survival to 59.3% (16 out of 27 mice). Similarly, combining HSCs with either activated T cells (55.6%, 15 out of 27 mice) or MSCs (50.0%, 15 out of 30 mice) yielded comparable outcomes. Most strikingly, co-transfusion of HSCs with both MSCs and activated T cells achieved an impressive survival rate of 82.8% (compared to HSCs alone, *p* = 0.0387 or HSCs with MSCs, *p* = 0.0061, Log-rank test) (Fig. 3C). These results underscore the synergistic potential of combining HSCs with MSCs and activated T cells to significantly enhance recipient survival rates.

### Synergistic augmentation of neutrophil reconstitution through co-transplantation of HSCs with MSCs and activated T cells in humanized mice

**Fig. 4:**
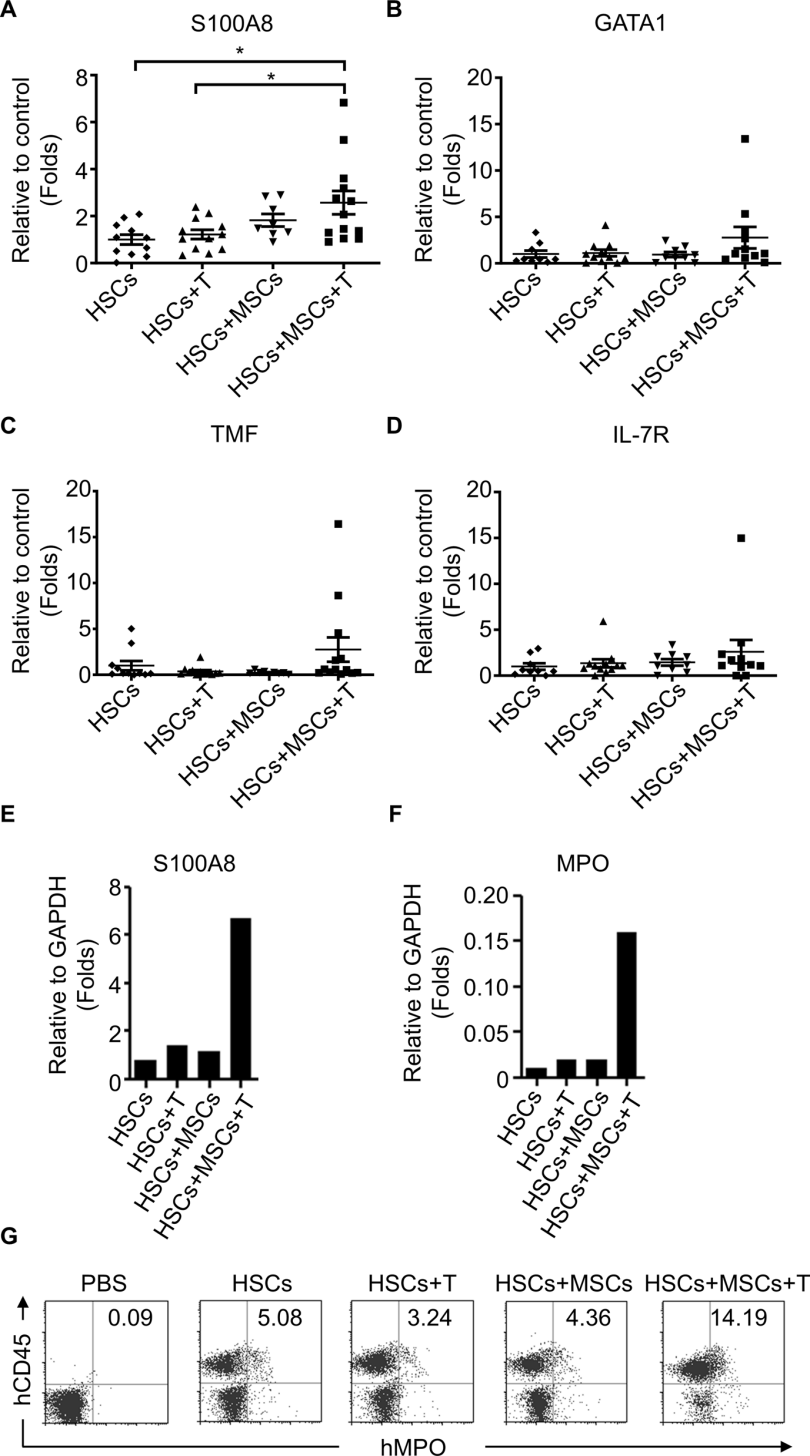
Co-transplantation of human HSCs with T cells and MSCs demonstrated granulocytic lineage development. Leukocytes were isolated from the peripheral blood of humanized NOD/SCID mice that were transplanted with various combinations of human cord blood HSCs (1 × 10^5^), MSCs (5 × 10^5^), and autologous T cells (1 × 10^5^) as indicated (HSCs: ◆, *n* = 11; HSCs + T: ▲, *n* = 12; HSCs + MSCs: ▼, *n* = 8; HSCs + MSCs + T: ■, *n* = 13). Total RNA was purified from the isolated leukocytes and analyzed by the real-time PCR to assess the mRNA levels of representative cell-lineage markers, including **A** S100A8 (Granulocyte), **B** GATA1 (Erythrocyte), **C** TMF1 (Megakaryocyte), and **D** IL7R (Lymphocyte). Additionally, bone marrow cells were harvested for evaluating the gene expression of human S100A8 (**E**) and MPO (**F**) by real-time PCR. **G** These bone marrow cells were also subjected to flow cytometry analysis to determine the expression of hCD45 and hMPO

We evaluated the efficiency of human immune cell lineage reconstitution in recipient mice co-transplanted with HSCs alone or in combination with MSCs, activated T cells, or both. The expression analysis of human S100A8, GATA1, TMF, and IL-7R mRNA served as markers for cells derived from granulocytic, erythrocytic, megakaryocytic, and lymphocytic lineages, respectively. Compared to mice receiving HSCs alone, those infused with HSCs, MSCs, and activated T cells displayed a modest increase in human GATA1, TMF, and IL-7R mRNA levels in peripheral white blood cells, though these differences were not statistically significant (Fig. 4B–D). Peripheral white blood cells from mice infused with HSCs/MSCs or HSCs/activated T cells showed low levels of human S100A8 mRNA. Notably, human S100A8 mRNA levels were significantly elevated in mice co-transplanted with HSCs, MSCs, and activated T cells compared to those receiving HSCs alone or with activated T cells (Fig. 4A, *p* = 0.0253 and *p* = 0.0124, respectively, unpaired two-tailed *t*-test), suggesting enhanced neutrophil lineage formation in this group.

To further explore the impact on neutrophil development, we analyzed bone marrow samples collected 21 weeks post-infusion. Mice receiving HSCs combined with MSCs and activated T cells exhibited significantly higher mRNA levels of human S100A8 and myeloperoxidase (MPO) from bone marrow compared to other groups (Fig. 4E and F). MPO, a key neutrophil marker, was expressed at higher levels in these mice than in those receiving HSCs alone or in combination with either MSCs or activated T cells. Flow cytometry revealed that human MPO-expressing cells in the bone marrow were also positive for human CD45 (Fig. 4G). Control mice receiving PBS showed no human MPO^+^CD45^+^ cells, while those receiving HSCs alone or in combination exhibited modest levels. However, mice co-transplanted with HSCs, MSCs, and activated T cells showed a significantly higher frequency (14.2%) of human MPO^+^CD45^+^ cells, indicating enhanced neutrophil reconstitution.

It is important to note that the use of a single batch of CD34^+^HSCs for cell transplantation limited the diversity of transplant combinations, affecting subsequent infection analyses. Additionally, the quantity and activity of reconstituted immune cells varied across transplant animals. Although data were collected from multiple batches, we present only one representative dataset for clarity in Fig. 4E–G. These findings highlight the synergistic effect of co-transplanting HSCs with MSCs and activated T cells in promoting neutrophil development in NOD-SCID mice.

### Augmented neutrophil reconstitution via co-transplantation of MSCs and T cells enhances anti-pseudomonal defense in HSC recipients

**Fig. 5:**
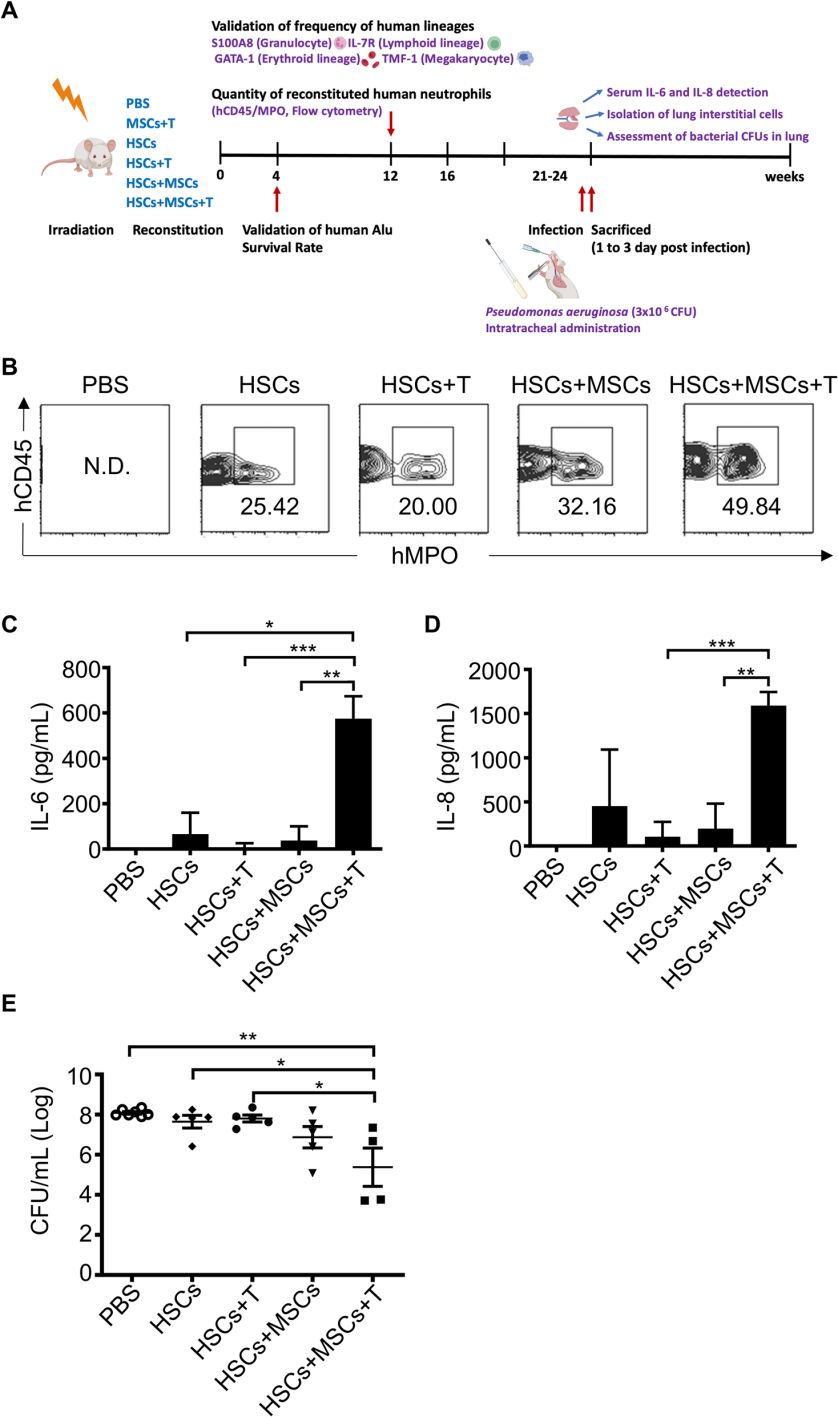
Enhanced anti-Pseudomonal defense via neutrophil reconstitution by co-transplantation of MSCs and T cells in recipients of human HSCs. **A** Experimental timeline. NOD/SCID mice were given sub-lethal irradiation and then transplanted with PBS (as negative control) or different combinations of human HSCs, MSCs, and T cells. At 4 and 12 weeks after transplantation, mice were assessed for human Alu signal and quantified for reconstituted human HSCs, respectively. Subsequently, after 21 weeks of HSC reconstitution, mice were anesthetized with freshly prepared 2% avertin (0.019 mL/g) via intraperitoneal injection, followed by infection with a half-lethal dose (LD50) (~ 3 × 10 ^6^ CFU, colony formation unit) of *Pseudomonas aeruginosa* for 1 day to induce acute pulmonary infection. Lung tissues were collected, and lung interstitial cells (mCD146^+^cells) were isolated and gated with hCD45 to determine the frequency of human CD45^+^MPO.^+^ cells (**B**). The expression levels of human IL-6 (**C**) and human IL-8 (**D**) in the sera collected from infected mice were examined via ELISA. **E** The lung tissues of infected groups were collected, disrupted with a tissue chopper, and plated on LB agar plates to calculate bacterial load (CFU). Data were expressed as mean ± S.E.M., and significant differences between groups were indicated (*p < 0.05, **p < 0.01; two-sided unpaired *t*-test, ns, not significant)

To investigate the physiological mechanisms underlying the role of MSCs and T cells in neutrophil development, we established an acute pulmonary infection model using *Pseudomonas aeruginosa* to assess neutrophil activity (Fig. 5A). Neutrophils are critical for bacterial clearance and host survival during *Pseudomonas aeruginosa* infection [23]. As anticipated, the group receiving HSCs co-transplanted with MSCs and activated T cells exhibited a significant increase in neutrophil reconstitution compared to the control group (Fig. 5B). Humanized mice co-transplanted with HSCs, MSCs, and T cells, and subsequently infected with *Pseudomonas aeruginosa*, demonstrated enhanced production of the anti-bacterial cytokines IL-6 (Fig. 5C, HSCs + MSCs + T compared to HSCs + MSCs, *p* = 0.0047; HSCs + MSCs + T compared to HSCs + T, *p* = 0.0002; HSCs + MSCs + T compared to HSCs, *p* = 0.034, unpaired two-tailed *t*-test) and IL-8 (Fig. 5D, HSCs + MSCs + T compared to HSCs + MSCs, *p* = 0.0087; HSCs + MSCs + T compared to HSCs + T, *p* = 0.0005; unpaired two-tailed *t*-test). Furthermore, quantitative analysis of bacterial load in infected pulmonary tissue revealed a significant reduction in bacterial burden in HSCs + MSCs + T-reconstituted mice, with significantly lower log CFU/mL than PBS (*p* = 0.0037), HSCs (*p* = 0.0415), and HSCs + T (*p* = 0.0259, unpaired two-tailed *t*-test) (Fig. 5E).

These findings suggest that the interaction between MSCs and T cells preferentially promotes HSC differentiation into neutrophils, thereby enhancing anti-bacterial immune responses.

## Discussion

Stem cell transplantation is a critical therapeutic intervention for treating a range of medical conditions, including cancer, congenital metabolic disorders, severe aplastic anemia, and immunodeficiency disorders. However, challenges such as graft failure and graft-versus-host disease (GvHD) continue to limit optimal efficacy and long-term survival [24]. Although current immunosuppressive therapies for GvHD can provide temporary relief, they often increase the risk of infection or tumor relapse by impairing anti-tumor immunity. As such, improving transplant success while reducing infection risk during immune reconstitution remains a key clinical priority. Addressing graft failure, GvHD, and infection susceptibility is essential to optimizing outcomes and ensuring the long-term survival of stem cell transplant recipients.

The immunomodulatory potential of MSCs has emerged as a promising therapeutic strategy in allogeneic transplant settings, particularly in mitigating GvHD and other immune-related complications. Enhancing the effectiveness of stem cell therapy by better harnessing the immunomodulatory properties of MSCs remains a central goal in clinical practice [25]. In our study, we assessed the impact of MSCs and/or activated T cells in combination with HSCs using ex vivo and co-transplantation in vivo models. While ex vivo analysis demonstrated considerable variability in HSC differentiation, co-culture with MSCs and activated T cells led to increased S100A8 expression, a neutrophil marker gene that was induced earlier and sustained over time (data not shown). Remarkably, we observed significantly improved survival rates in mice co-transfused with MSCs, activated T cells, and HSCs compared to those receiving HSCs alone or in combination with either MSCs or activated T cells. Co-transfused mice demonstrated enhanced neutrophil recovery and greater resistance to bacterial infections, underscoring the therapeutic potential of combining MSCs and activated T cells with HSCs to improve immune reconstitution and infection defense.

The success of cell transplantation is influenced by several factors, including the availability and quantity of HSCs, the condition of the bone marrow niche, and the immune status of the recipient [26–28]. A key challenge is that the quantity of HSCs from a single source is often limited, and expansion techniques can compromise potency, leading to suboptimal transplantation outcomes. Alternative strategies, including the use of cytokines to stimulate stem cell production and targeting nuclear hormone receptors, have been explored to enhance transplantation efficacy [29]. Nonetheless, navigating complex regulatory mechanisms and individual variability remains challenging. MSCs are considered major contributors to the bone marrow HSC niche [30], and several studies have shown that MSC transplantation can enhance HSC engraftment in both animal models [31] and human subjects [32]. Our previous research demonstrated that MSCs induce activated CD4^+^CD45RO^+^ memory T cells to produce interleukin-17 (IL-17), a cytokine essential for neutrophil differentiation, activation, and recruitment [18, 21]. Additionally, IL-17 has been shown to mobilize HSCs with both short- and long-term repopulating ability [33]. In line with these findings, although co-transfusion of MSCs with HSCs into immunodeficient mice did not significantly improve HSCs engraftment, it did lead to increased neutrophil frequencies in peripheral blood (Fig. 4A) and bone marrow (Fig. 4G) when combined with activated T cells. This suggests that the interaction between MSCs and T cells promotes hematopoiesis toward granulocyte development, facilitating neutrophil recovery in HSCT recipients. Our findings underscore the potential of MSCs and activated T cells in mediating effective neutrophil recovery post-transplantation, and highlight their crucial role in enhancing HSC engraftment by providing essential cytokines and growth factors in the hematopoietic microenvironment. While the xenogeneic/allogeneic stem cell transplantation model used in this study may not perfectly reflect human hematopoiesis, it remains a valuable tool for understanding stem cell engraftment dynamics, tracking human immune cell dissemination, and evaluating pathogen resistance.

Neutrophils represent a pivotal immune cell population that rapidly repopulates following allogeneic HSCT, serving as a critical component of early immune system recovery. Despite their importance, the intricate functional and phenotypic characteristics of neutrophil subsets emerging during post-transplantation reconstitution remain largely enigmatic [34–36]. Research has revealed neutrophils as a remarkably heterogeneous cell population with dual capabilities: they can either exacerbate GvHD or demonstrate regulatory potential in modulating inflammatory responses, a complexity exemplified by LCN2-positive (LCN2^+^) neutrophils [37, 38]. Intriguingly, *Lcn2* gene expression also significant increased while naive CD4^+^ T cells in vitro differentiation into Th17, Th1, and Th2 cells [39]. In our comprehensive ex vivo co-cultured experiments demonstrated LCN2 expression across different cellular contexts—including HSCs alone, T cells, and HMT conditions—with the HMT setting revealing the most pronounced expression levels (Fig. 2B). This observation suggests a potential synergistic cellular interaction that might facilitate the development of a protective neutrophil phenotype. Emerging clinical research provides additional context for LCN2’s significance. Studies in renal transplantation have demonstrated that neutrophil gelatinase-associated lipocalin (NGAL) levels in patient urine and serum can serve as a powerful predictive marker for early graft function recovery [40, 41]. The multifaceted nature of LCN2 gene expression—spanning its implications across neutrophil subsets, its regulatory roles in transplantation and cell therapies, and its antimicrobial capabilities—represents a fertile ground for further scientific investigation. Notably, the potential of LCN2 in enhancing and modulating T cell-based therapies is particularly promising. By offering deeper insights into cellular interactions and immune responses, this line of research could potentially revolutionize our approach to therapeutic strategies, ultimately leading to improved clinical outcomes in transplantation and immunotherapy.

It is important to note that the stem cell source used in this study was umbilical cord blood (CB), whereas many autologous transplant recipients receive granulocyte-colony stimulating factor (G-CSF)-mobilized peripheral blood stem cells (PBSCs). Beyond its role in stem cell mobilization, G-CSF is also crucial in treating neutropenia [42, 43]. The profound heterogeneity observed among repopulating neutrophil subpopulations implies multifaceted roles in orchestrating the intricate immunological processes driving early engraftment. A comprehensive understanding of the nuanced effector functions, developmental paths, and immunoregulatory capacities in this emerging neutrophil landscape is imperative to delineate their contributions to therapeutic outcomes and potential complications. Future studies that systematically analyze neutrophil activity and subset distributions in various tissues following transplantation with G-CSF-mobilized PBSCs or allogeneic grafts will be critical for optimizing the therapeutic potential of these cells, particularly in combination with MSCs and activated T cells.

Chimeric antigen receptor (CAR) T-cell therapy, an advanced cancer treatment, faces challenges such as severe and prolonged cytopenias post-infusion, which hinder its effectiveness. Stem cell augmentation is emerging as a promising solution to address these post-CAR T-cell cytopenias, providing crucial support during immune reconstitution [11, 44]. Enhancing the generation of CAR T memory stem cells, which could improve the long-term persistence and self-renewal of T cells, is another potential avenue for improving therapeutic outcomes [45]. In our study, we explored the effects of MSCs and T cells on HSC transplantation, focusing on neutrophil recovery as a critical determinant of survival. Although extended monitoring of memory T cells post-transplantation was not feasible, future studies should investigate the potential benefits of co-infusion of MSCs and HSCs during CAR T-cell therapy, particularly in mitigating complications such as cytokine release syndrome (CRS) and immune effector cell-associated neurotoxicity syndrome (ICANS) and hematologic toxicity associated with CAR T treatment. Systematic implementation and interrogation of such combinatorial cell therapy approaches in clinical settings hold considerable promise for enhancing immune reconstitution and improving outcomes for transplant recipients. Comprehensive studies delineating the mechanisms by which MSCs and either allo- or autologous HSCs synergize to modulate CAR T-cell subset distributions, effector functions, and persistence will be vital for realizing the full therapeutic potential of these strategies.

Advancements in the preparation and storage of MSCs and immune cells, along with improvements in CAR T-cell modification, hold significant promise for the future of stem cell transplantation. Utilizing MSCs to mitigate allogeneic cell rejection while enhancing autologous T cells as allies in HSC transplantation presents a promising avenue for improving outcomes. Our findings emphasize the substantial impact of MSCs and activated T cells in enhancing HSCT outcomes, particularly in promoting neutrophil recovery and strengthening immune defenses against infection.

## Limitations

This study underscores the potential of combining MSCs, activated T cells, and CB-derived HSCs to enhance neutrophil reconstitution and improve survival post-transplantation. However, several limitations must be addressed. The reliance on CB-derived HSCs constrains the generalizability of findings to other stem cell sources, such as G-CSF-mobilized peripheral blood stem cells. A key question remains whether G-CSF-treated HSCs can still enhance neutrophil generation when combined with MSCs and autologous T cells, warranting further investigation. Additionally, the use of immunodeficient NOD/SCID and NOG mouse models fails to fully replicate human immune dynamics or accurately assess complications such as GvHD. Variability in reconstitution efficiency across batches, along with a predominant focus on neutrophils over other immune lineages, narrows the scope of findings. Moreover, the short-term follow-up limits the ability to evaluate long-term immune reconstitution, while inconsistencies in the ex vivo co-culture system emphasize the need for standardized protocols. Furthermore, optimizing HSC-based therapies—including determining ideal cell ratios and maximizing synergistic effects—requires extensive clinical data collection and analysis. A more comprehensive understanding is essential to tailor these treatments effectively for patients with diverse medical needs. To address these challenges, future research should prioritize more clinically relevant models and human trials to validate and refine this approach, ultimately paving the way for its broader therapeutic application.

## Conclusions

Our findings indicate that co-transplantation of HSCs with MSCs and activated T cells may represent a promising strategy to enhance engraftment, suggesting a potential paradigm shift in cell transplantation. These results offer important insights into the therapeutic potential of combining HSCs with MSCs and activated T cells for clinical application.

## Data Availability

Data is provided within the manuscript and is available on reasonable request.

## Abbreviations

HSCT: Hematopoietic stem cell transplantation
MSCs: Mesenchymal stem cells
HSCs: Hematopoietic stem cells
GvHD: Graft-versus-host disease
IL-17: Interleukin-17
MPO: Myeloperoxidase
PBMCs: Human peripheral blood mononuclear cells
Ct: Cycle threshold
GAPDH: Glyceraldehyde-3-phosphate dehydrogenase
S100A8: S100 calcium binding protein A8
NOD/SCID: NOD.CB17-Prkdc^scid^/Tcu
NOG: NOD/Shi-scid IL2Rgamma(null)
ELISA: Enzyme-linked immunosorbent assay
CFUs: Colony-forming units
IT: Intra-tracheal
FACS: Fluorescence-activated cell sorting
HMT: HSCs, MSCs, and T cells
NOX2: NADPH oxidase 2
MMP-9: Matrix metalloproteinase-9
LCN2: Lipocalin-2
NGAL: Neutrophil gelatinase-associated lipocalin
CB: Cord blood
G-CSF: Granulocyte-colony stimulating factor
PBSCs: Peripheral blood stem cells
CAR: Chimeric antigen receptor
CRS: Cytokine release syndrome
ICANS: Immune effector cell-associated neurotoxicity syndrome

## References

[1] Orkin SH, Zon LI. Hematopoiesis: an evolving paradigm for stem cell biology. Cell. 2008;132(4):631–44. 18295580 10.1016/j.cell.2008.01.025 PMC2628169

[2] Crane GM, Jeffery E, Morrison SJ. Adult haematopoietic stem cell niches. Nat Rev Immunol. 2017;17(9):573–90. 28604734 10.1038/nri.2017.53

[3] Copelan EA. Hematopoietic stem-cell transplantation. N Engl J Med. 2006;354(17):1813–26. 16641398 10.1056/NEJMra052638

[4] Adams GB, Martin RP, Alley IR, Chabner KT, Cohen KS, Calvi LM, Kronenberg HM, Scadden DT. Therapeutic targeting of a stem cell niche. Nat Biotechnol. 2007;25(2):238–43. 17237769 10.1038/nbt1281

[5] Calvi LM, Adams GB, Weibrecht KW, Weber JM, Olson DP, Knight MC, Martin RP, Schipani E, Divieti P, Bringhurst FR, et al. Osteoblastic cells regulate the haematopoietic stem cell niche. Nature. 2003;425(6960):841–6. 14574413 10.1038/nature02040

[6] Mendez-Ferrer S, Michurina TV, Ferraro F, Mazloom AR, Macarthur BD, Lira SA, Scadden DT, Ma’ayan A, Enikolopov GN, Frenette PS. Mesenchymal and haematopoietic stem cells form a unique bone marrow niche. Nature. 2010;466(7308):829–34. 20703299 10.1038/nature09262 PMC3146551

[7] Zhou J, Shi Y. Mesenchymal stem/stromal cells (MSCs): origin, immune regulation, and clinical applications. Cell Mol Immunol. 2023;20(6):555–7. 37225837 10.1038/s41423-023-01034-9 PMC10229593

[8] Jiang W, Xu J. Immune modulation by mesenchymal stem cells. Cell Prolif. 2020;53(1):e12712. 31730279 10.1111/cpr.12712 PMC6985662

[9] Markov A, Thangavelu L, Aravindhan S, Zekiy AO, Jarahian M, Chartrand MS, Pathak Y, Marofi F, Shamlou S, Hassanzadeh A. Mesenchymal stem/stromal cells as a valuable source for the treatment of immune-mediated disorders. Stem Cell Res Ther. 2021;12(1):192. 33736695 10.1186/s13287-021-02265-1 PMC7971361

[10] Lopez-Santalla M, Bueren JA, Garin MI. Mesenchymal stem/stromal cell-based therapy for the treatment of rheumatoid arthritis: an update on preclinical studies. EBioMedicine. 2021;69:103427. 34161884 10.1016/j.ebiom.2021.103427 PMC8237294

[11] Sharma N, Reagan PM, Liesveld JL. Cytopenia after CAR-T cell therapy-a brief review of a complex problem. Cancers (Basel). 2022;14(6):1501–11. 10.3390/cancers14061501 PMC8946106 35326654

[12] Ba Y, Shi Y, Jiang W, Feng J, Cheng Y, Xiao L, Zhang Q, Qiu W, Xu B, Xu R, et al. Current management of chemotherapy-induced neutropenia in adults: key points and new challenges: Committee of Neoplastic Supportive-Care (CONS), China Anti-Cancer Association Committee of Clinical Chemotherapy, China Anti-Cancer Association. Cancer Biol Med. 2020;17(4):896–909. 33299642 10.20892/j.issn.2095-3941.2020.0069 PMC7721096

[13] Kao RL, Holtan SG. Host and graft factors impacting infection risk in hematopoietic cell transplantation. Infect Dis Clin North Am. 2019;33(2):311–29. 30940461 10.1016/j.idc.2019.02.001

[14] Ferdjallah A, Young JH, MacMillan ML. A review of infections after hematopoietic cell transplantation requiring PICU care: transplant timeline is key. Front Pediatr. 2021;9:634449. 34386464 10.3389/fped.2021.634449 PMC8353083

[15] Vento S, Cainelli F. Infections in patients with cancer undergoing chemotherapy: aetiology, prevention, and treatment. Lancet Oncol. 2003;4(10):595–604. 14554236 10.1016/s1470-2045(03)01218-x

[16] Thursky KA, Worth LJ. Can mortality of cancer patients with fever and neutropenia be improved? Curr Opin Infect Dis. 2015;28(6):505–13. 26374951 10.1097/QCO.0000000000000202

[17] Lyman GH. Impact of chemotherapy dose intensity on cancer patient outcomes. J Natl Compr Canc Netw. 2009;7(1):99–108. 19176210 10.6004/jnccn.2009.0009

[18] Hsu SC, Wang LT, Yao CL, Lai HY, Chan KY, Liu BS, Chong P, Lee OK, Chen HW. Mesenchymal stem cells promote neutrophil activation by inducing IL-17 production in CD4+ CD45RO+ T cells. Immunobiology. 2013;218(1):90–5. 22464815 10.1016/j.imbio.2012.02.007

[19] Varghese DS, Parween S, Ardah MT, Emerald BS, Ansari SA. Effects of aminoglycoside antibiotics on human embryonic stem cell viability during differentiation in vitro. Stem Cells Int. 2017;2017:2451927. 29147115 10.1155/2017/2451927 PMC5632925

[20] Yang YS, Huang TW, Huang YC, Huang WC, Hsu SY, Wu HC, Chen FJ, Shang HS, Sytwu HK, Kuo SC. In vitro and in vivo efficacy of minocycline-based therapy for Elizabethkingia anophelis and the impact of reduced minocycline susceptibility. Int J Antimicrob Agents. 2022;60(5–6):106678. 36184015 10.1016/j.ijantimicag.2022.106678

[21] Kolls JK, Linden A. Interleukin-17 family members and inflammation. Immunity. 2004;21(4):467–76. 15485625 10.1016/j.immuni.2004.08.018

[22] Krstic A, Mojsilovic S, Jovcic G, Bugarski D. The potential of interleukin-17 to mediate hematopoietic response. Immunol Res. 2012;52(1–2):34–41. 22392050 10.1007/s12026-012-8276-8

[23] Curran CS, Bolig T, Torabi-Parizi P. Mechanisms and targeted therapies for Pseudomonas aeruginosa lung infection. Am J Respir Crit Care Med. 2018;197(6):708–27. 29087211 10.1164/rccm.201705-1043SO PMC5855068

[24] Malard F, Holler E, Sandmaier BM, Huang H, Mohty M. Acute graft-versus-host disease. Nat Rev Dis Primers. 2023;9(1):27. 37291149 10.1038/s41572-023-00438-1

[25] Kadri N, Amu S, Iacobaeus E, Boberg E, Le Blanc K. Current perspectives on mesenchymal stromal cell therapy for graft versus host disease. Cell Mol Immunol. 2023;20(6):613–25. 37165014 10.1038/s41423-023-01022-z PMC10229573

[26] Sugiyanto M, Gosal S, Kosim A, Tahapary DL, Sianipar IR. Impact of the source of hematopoietic stem cells on immune reconstitution after transplantation: a systematic review. Eur J Haematol. 2023;111(1):4–14. 36950969 10.1111/ejh.13966

[27] Tang X, Wang Z, Wang J, Cui S, Xu R, Wang Y. Functions and regulatory mechanisms of resting hematopoietic stem cells: a promising targeted therapeutic strategy. Stem Cell Res Ther. 2023;14(1):73. 37038215 10.1186/s13287-023-03316-5 PMC10088186

[28] Ando T, Tachibana T, Tanaka M, Suzuki T, Ishiyama Y, Koyama S, Ogusa E, Numata A, Matsumoto K, Kanamori H, et al. Impact of graft sources on immune reconstitution and survival outcomes following allogeneic stem cell transplantation. Blood Adv. 2020;4(2):408–19. 31990335 10.1182/bloodadvances.2019001021 PMC6988395

[29] Guo B, Huang X, Broxmeyer HE. Enhancing human cord blood hematopoietic stem cell engraftment by targeting nuclear hormone receptors. Curr Opin Hematol. 2018;25(4):245–52. 29608487 10.1097/MOH.0000000000000429 PMC6152906

[30] Ehninger A, Trumpp A. The bone marrow stem cell niche grows up: mesenchymal stem cells and macrophages move in. J Exp Med. 2011;208(3):421–8. 21402747 10.1084/jem.20110132 PMC3058583

[31] Garrigos MM, de Oliveira FA, Nucci MP, Nucci LP, Alves ADH, Dias OFM, Gamarra LF. How mesenchymal stem cell cotransplantation with hematopoietic stem cells can improve engraftment in animal models. World J Stem Cells. 2022;14(8):658–79. 36157912 10.4252/wjsc.v14.i8.658 PMC9453272

[32] Crippa S, Bernardo ME. Mesenchymal stromal cells: role in the BM niche and in the support of hematopoietic stem cell transplantation. Hemasphere. 2018;2(6):e151. 31723790 10.1097/HS9.0000000000000151 PMC6745957

[33] Schwarzenberger P, Huang W, Ye P, Oliver P, Manuel M, Zhang Z, Bagby G, Nelson S, Kolls JK. Requirement of endogenous stem cell factor and granulocyte-colony-stimulating factor for IL-17-mediated granulopoiesis. J Immunol. 2000;164(9):4783–9. 10779785 10.4049/jimmunol.164.9.4783

[34] Majhail NS, Farnia SH, Carpenter PA, Champlin RE, Crawford S, Marks DI, Omel JL, Orchard PJ, Palmer J, Saber W, et al. Indications for autologous and allogeneic hematopoietic cell transplantation: guidelines from the American Society for Blood and Marrow Transplantation. Biol Blood Marrow Transplant. 2015;21(11):1863–9. 26256941 10.1016/j.bbmt.2015.07.032 PMC4830270

[35] Ley K, Hoffman HM, Kubes P, Cassatella MA, Zychlinsky A, Hedrick CC, Catz SD. Neutrophils: new insights and open questions. Sci Immunol. 2018;3(30):eaat4579. 30530726 10.1126/sciimmunol.aat4579

[36] Ramaprasad C, Pouch S, Pitrak DL. Neutrophil function after bone marrow and hematopoietic stem cell transplant. Leuk Lymphoma. 2010;51(5):756–67. 20350278 10.3109/10428191003695678

[37] Schwab L, Goroncy L, Palaniyandi S, Gautam S, Triantafyllopoulou A, Mocsai A, Reichardt W, Karlsson FJ, Radhakrishnan SV, Hanke K, et al. Neutrophil granulocytes recruited upon translocation of intestinal bacteria enhance graft-versus-host disease via tissue damage. Nat Med. 2014;20(6):648–54. 24836575 10.1038/nm.3517

[38] Czech M, Schneider S, Peltokangas N, El Khawanky N, Ghimire S, Andrieux G, Hulsdunker J, Krausz M, Proietti M, Braun LM, et al. Lipocalin-2 expression identifies an intestinal regulatory neutrophil population during acute graft-versus-host disease. Sci Transl Med. 2024;16(735):eadi1501. 38381845 10.1126/scitranslmed.adi1501

[39] Lee SA, Noel S, Kurzhagen JT, Sadasivam M, Pierorazio PM, Arend LJ, Hamad AR, Rabb H. CD4(+) T cell-derived NGAL modifies the outcome of ischemic acute kidney injury. J Immunol. 2020;204(3):586–95. 31889023 10.4049/jimmunol.1900677 PMC6981061

[40] Cappuccilli M, Capelli I, Comai G, Cianciolo G, La Manna G. Neutrophil gelatinase-associated lipocalin as a biomarker of allograft function after renal transplantation: evaluation of the current status and future insights. Artif Organs. 2018;42(1):8–14. 29266311 10.1111/aor.13039 PMC5814881

[41] Goldstein SL. Acute kidney injury biomarkers: renal angina and the need for a renal troponin I. BMC Med. 2011;9:135. 22189039 10.1186/1741-7015-9-135 PMC3287120

[42] Thunstrom Salzer A, Niemiec MJ, Hosseinzadeh A, Stylianou M, Astrom F, Rohm M, Ahlm C, Wahlin A, Ermert D, Urban CF. Assessment of neutrophil chemotaxis upon G-CSF treatment of healthy stem cell donors and in allogeneic transplant recipients. Front Immunol. 2018;9:1968. 30254629 10.3389/fimmu.2018.01968 PMC6141688

[43] Theyab A, Algahtani M, Alsharif KF, Hawsawi YM, Alghamdi A, Alghamdi A, Akinwale J. New insight into the mechanism of granulocyte colony-stimulating factor (G-CSF) that induces the mobilization of neutrophils. Hematology. 2021;26(1):628–36. 34494505 10.1080/16078454.2021.1965725

[44] Jogalekar MP, Rajendran RL, Khan F, Dmello C, Gangadaran P, Ahn BC. CAR T-Cell-Based gene therapy for cancers: new perspectives, challenges, and clinical developments. Front Immunol. 2022;13:925985. 35936003 10.3389/fimmu.2022.925985 PMC9355792

[45] Guo A, Huang H, Zhu Z, Chen MJ, Shi H, Yuan S, Sharma P, Connelly JP, Liedmann S, Dhungana Y, et al. cBAF complex components and MYC cooperate early in CD8(+) T cell fate. Nature. 2022;607(7917):135–41. 35732731 10.1038/s41586-022-04849-0 PMC9623036

